# Nine-day continuous recording of EEG and 2-hour of high-density EEG under chronic sleep restriction in mice

**DOI:** 10.1038/s41597-022-01354-x

**Published:** 2022-05-23

**Authors:** Hio-Been Han, Bowon Kim, Youngsoo Kim, Yong Jeong, Jee Hyun Choi

**Affiliations:** 1 Center for Neuroscience, Korea Institute of Science and Technology (KIST), 5 Hwarang-ro 14-gil, Seongbuk-gu, Seoul, 02792 Republic of Korea; 2 Program of Brain and Cognitive Engineering, Korea Advanced Institute of Science and Technology, Daejeon, 34141 Republic of Korea; 3 Department of Chemistry, University of Michigan, 930 N. University Ave., Ann Arbor, MI 48109-1055 United States; 4 Department of Bio and Brain Engineering, Korea Advanced Institute of Science and Technology, Daejeon, 34141 Republic of Korea; 5 Department of Neural Sciences, University of Science and Technology, 217, Gajeong-ro, Yuseong-gu, Daejeon, 34113 Republic of Korea

**Keywords:** Sleep deprivation, Sleep, Dynamical systems

## Abstract

This work provides an EEG dataset collected from nine mice during the sleep deprivation (SD) paradigm for the sleep science community. It includes 9-day of continuous recording of the frontal and parietal EEG, accelerometer, and 2-hour of high-density EEG (HD-EEG) under SD and SD-free conditions. Eighteen hours of SD were conducted on 5 consecutive days. The HD-EEG data were saved in the EEGLAB format and stored as the brain imaging data structure (BIDS). These datasets can be used to (i) compare mouse HD-EEG to human HD-EEG, (ii) track oscillatory activities of the sleep EEG (e.g., slow waves, spindles) across the cortical regions under different conditions of sleep pressure, and (iii) investigate the cortical traveling waves in the mouse brain. We also provided Python code for basic analyses of this dataset, including the detection of slow waves and sleep spindles. We hope that our dataset will reveal hidden activities during sleep and lead to a better understanding of the functions and mechanisms of sleep.

## Abstract (summary)

**Table:** 

Measurement(s)	electroencephalography (EEG)
Technology Type(s)	electroencephalography (EEG)
Factor Type(s)	Chronic sleep restriction
Sample Characteristic - Organism	Mus musculus

## Background & Summary

Sleep deprivation (SD), i.e., experimentally induced sleep loss, is a widely used experimental procedure to unveil various aspects of sleep functions. Investigations of SD-induced impairments in general physiology and cognitive functions have provided a foundation for understanding sleep functions. In recent decades, introduction of genetically modified animals (mostly rodents) has allowed a more comprehensive understanding of sleep functions at the molecular, synaptic, neuronal, and cognitive level. SD approaches in rodents have provided a mechanistic understanding of sleep function (for a review, see^1^), and yielded potential drug targets for sleep regulation (for a review, see^2^).

It is noteworthy that the key effect of SD is an increase in sleep pressure (i.e., sleepiness). The more deviant the individual is from the homeostatic equilibrium state, the higher the sleep pressure induced in the individual^3^. Thus, SD time is considered as an index of sleep pressure, and the influence of SD differs according to the duration and repeatability of SD periods. For instance, a prolonged continuous SD revealed the vital role of sleep in life of rats^4^, whereas partial SD studies suggested the beneficial nature of sleep intervention for therapeutic purposes^5^. In contrast, when SD repeats, devastating effects have been observed in memory, emotion regulation, immunity, and general health, as experienced by insomnia patients (for reviews, see^6,7^).

SD is also associated with alterations in EEG rhythms and behaviors. Slow wave activity and spindles during NREM sleep have with physiological relevance, such as facilitating glymphatic function^8^ and adenosine washout^9^, and cognitive relevance such as motor performance^10^ and memory consolidation^11–13^, and these relevance have been well documented in the literature. Likewise, the role of theta rhythms in memory consolidation is manifested by REM-specific sleep deprivation techniques^14^. All these findings suggest that sleep is not simple, but rather a temporally and spatially complex state of the brain. Decomposing the spatial variations of EEG rhythms can be a gateway to understanding the brain and building a decoder for a map linking the spatio-temporal-frequency EEG patterns with relevant specific functions. In this aspect, conducting an HD-EEG may be advantageous, as it permits the reconstruction of a topographical map of individual rhythms.

Here, we provide the frontal and parietal EEG dataset recorded continuously for 9 days in mice, where 18 hours of SD per day was conducted from the 2nd to 5th day of continuous recording. We also provide the HD-EEG (36 channels) dataset during 2 hours of sleep after sleep restriction, which was recorded together with the frontal and parietal EEG. The days in the dataset are the day before SD, the 1st, 3rd, and 5th day of SD, and the 1st and 3rd days after SD. The number of mice in this dataset was nine. The mice were unrestricted during hours of sleep opportunity, and they were placed in a motorized wheel to disturb mice every 2–3 s for SD. We also provide sleep stage label data, which were classified into three stages (i.e., wake, NREM, or REM) in every 10 s of the EEG data. An accelerometer (2 G) was used instead of electromyography to minimize surgical harm, and raw traces are included in the dataset. Parts of the data have already been used to characterize the influence of repeated SD. Our previous works focused on analyzing the topographical changes of EEG rhythms during NREM^15^ and REM sleep^16^, which have revealed different influences of SD in different EEG rhythms across the cortices.

The mouse HD-EEG in this dataset was acquired with a polyimide-based microarray placed on the skull^17^ (see the video in^18^). The spatial distribution of the mouse HD-EEG can be compared to that of the human HD-EEG, providing comparative neuroimaging between the two species. We have proven the use of mouse HD-EEG for retrieving local and global cortical activities, including the somatosensory and motor cortex^19^, prefrontal cortex^1^, anterior versus posterior spindles^20^, and global versus local seizures^21^. Among these studies, we previously provided a full dataset of evoked auditory stimulation under time-locked optogenetic stimulation^22^. In our previous work, we provided the Python code for topographical mapping as well as the code for observing evoked potentials and spectrograms, which are routine analyses in the EEG study. Here, we provide the Python codes for further basic analyses, including the detection of sleep-related activities (e.g., slow wave activity, spindles).

## Methods

The detailed experimental procedures of surgery, data acquisition, and detailed data analyses for sleep stage classification and preprocessing are described in our previous works^15,16^.

### Animals

Nine male mice of C57BL/6 and 129S4/SvJae hybrid varieties (12 weeks old at the time of surgery) were used in the study. Animals were maintained on a 12-hours of light and dark cycle (light on at 8 AM) with freely available food and water. All animal experimental procedures were approved by the Institutional Animal Care and Use Committee at Korea Institute of Science and Technology (AP-2014L7002).

### Mouse EEG electrodes

**Fig. 1:**
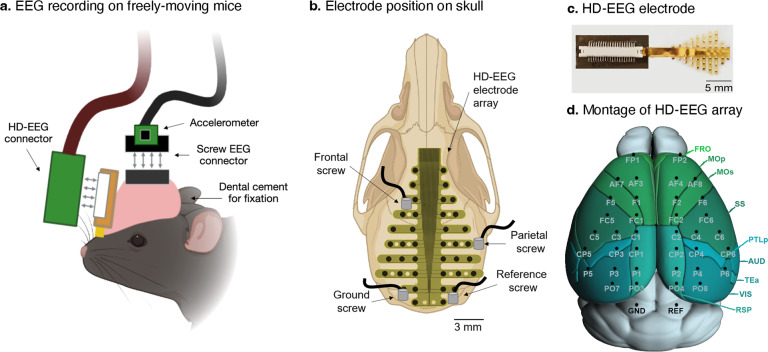
Recording of EEG. (**a**) Schematic illustration of the recording head-stages on a mouse head. (**b**) The position of electrodes are marked on an illustration of a mouse skull. A total of 42 (4 screws and 38 HD-EEG arrays) electrodes were placed, and 38 channels (2 screws and 36 HD-EEG arrays) were used for data acquisition. (**c**) Example photograph of the HD-EEG electrode array. (**d**) Channel montage of the HD-EEG array.

A schematic diagram of the EEG application is shown in Fig. 1a. Screw and HD-EEG electrodes were secured together on a skull using dental cements (Vertex-Dental, Zeist, Netherlands). We used custom-made screw electrodes using a stainless tapping screw (0.8 mm, Nitto Seiko Co., Japan, impedance: 10–50 kΩ at 1 kHz) for conventional two-channel EEG. The application of the screw EEG and HD-EEG is shown in Fig. 1b. The position of frontal EEG was 1.5 mm anteroposterior and 2 mm mediolateral, while parietal EEG was 2 mm anteroposterior and 4 mm mediolateral, relative to the bregma. For HD-EEG, we used a polyimide-based microarray (40 leads, thickness = 8 μm, width × length = 10 mm × 12.6 mm), as shown in Fig. 1c. The impedance of the electrode contact was approximately 100 kΩ at 1 kHz^17^, and the radius of the electrode was 500 μm. The center-to-center distance between the neighboring area was 1.2–2.0 mm. The numbering of HD-EEG electrodes and their relevant cortical areas are depicted in Fig. 1d. The mouse brain montage with cortical sub-regions was modified from the Allen Mouse Brain Atlas^23^ (Available from: brain-map.org/api/index.html).

### Noninvasive movement sensor

We used an accelerometer to sense the animal’s movement noninvasively. The sensitivity and dynamic range of the accelerometer were determined using a treadmill walking test^24^. The 2 G accelerometer (KXTC9–2050, Kionix Inc., Ithaca, NY, USA) has six axes (three for linear acceleration and three for gyroscope); however, we used one axis here for tracking the motion. The accelerometer was attached to the electrode connector so that the recording axis was orthogonal to the midline of the head of the mouse (Fig. 1a).

### Data acquisition

**Fig. 2:**
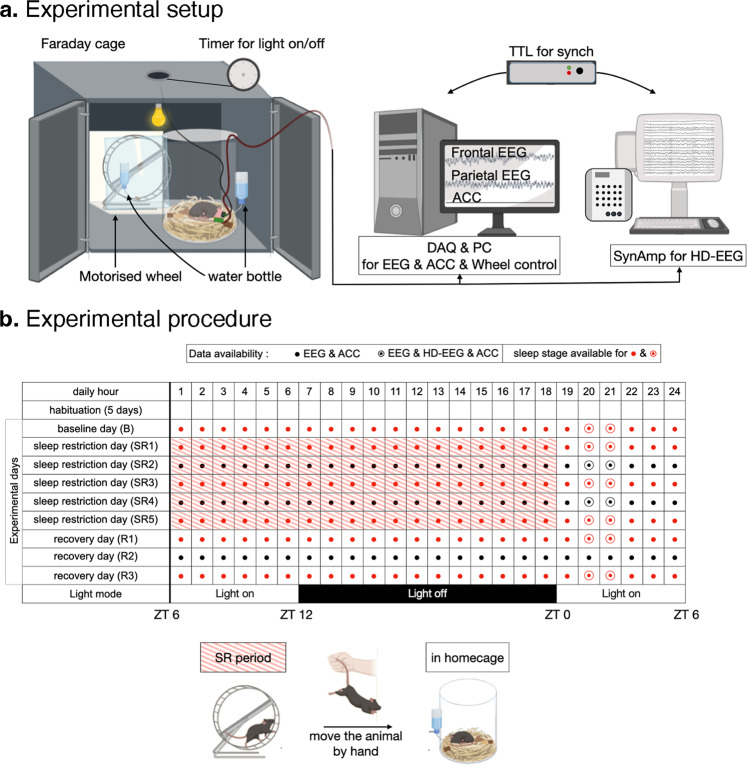
Experimental method. (**a**) Schematic illustration of the recording environment, composed of a chamber (left) and recording systems (right). (**b**) Table of experimental procedure describing the data availability. The shape of the dot denotes the availability of HD-EEG (red: available, black: screw EEG & Accelerometer data only). The color of the dot denotes the availability of sleep stage data (red: available, black: not labeled). Red shading indicates sleep restriction (SR) on a motorized wheel.

The experimental setup is shown in Fig. 2a. All recordings were conducted inside a sound-proof Faraday cage in a 12:12 light:dark cycle. We used a separate computer for HD-EEG to avoid recording data overflow. The frontal and parietal EEGs were acquired in a bipolar scheme referenced to a reference electrode on the interparietal bone with a ground electrode on the opposite side of the interparietal bone (see Fig. 1d) via an analog amplifier (QP511, Grass Technologies, West Warwick, RI, USA). These signals were then digitized with an analog-digital converter (Digidata 1440A, Molecular Devices, Sunnyvale, CA, USA) at a sampling frequency of 500 Hz, with a high-pass filter at 0.3 Hz, low-pass filter at 100 Hz, and 60 Hz hardware notch filter applied. The accelerometer shared an analog-digital converter. The HD-EEG was acquired via a Synamps 2 amplifier and a SCAN 4.5 data acquisition system (CompuMedics USA, Charlotte, NC, USA) at a 1 kHz sampling rate with a 0.1–100 Hz band-pass filter. The HD-EEG signal was down-sampled to 500 Hz after recording. The EEG and accelerometer were recorded continuously, and HD-EEG was recorded for 2 h only once to avoid saturation caused by polarization-induced DC drift, which is a common problem of the electrodes.

### Experimental paradigm

The overall schedule of the recording and its data availability are shown in Fig. 2b. In brief, after 5 days of habituation, a 9-day recording was conducted. From the 2^nd^ to the 6^th^ day, the mouse was placed on a motorized wheel (14 cm in diameter, 5.8 cm in width, Lafayette Instrument, IN, USA) whose side was blocked by a transparent wall for sleep restriction. The wheel moved at an approximate speed of 2.3 RPM for 4 s with a 2 s interval to disturb the mouse and it was scheduled to operate from ZT6 to ZT0. The dimensions of the sleep opportunity cage were 20 cm in diameter and 25 cm in height, and it was made of transparent acrylic. During the experiment, mice were continuously tethered to a recording wire for screw EEG. Furthermore, mice were not disturbed except when they had to move between the cage and wheel and when tethering was required for HD-EEG. They had free access to food and water.

## Data Records

### General description

The data availability is shown in Fig. 2b. In brief, there are three types of data: (i) continuous recording of EEG and accelerometer (24 h, 9 days), (ii) sleep scoring (24 hours, 6 days), and (iii) HD-EEG (2 hours, 8 days). All data are organized in the Brain Imaging Data Structure (BIDS) format^25,26^ and available in G-Node’s GIN repository^27^ (https://gin.g-node.org/hiobeen/Mouse_EEG_ChronicSleepRestriction_Kim_et_al).

Because of the large size of the dataset (almost 100 GB), we recommend using the gin-cli download tool rather than manual file-by-file downloading, following the GIN’s instruction (https://gin.g-node.org/G-Node/Info/wiki/GIN + CLI + Usage + Tutorial).

**Fig. 3:**
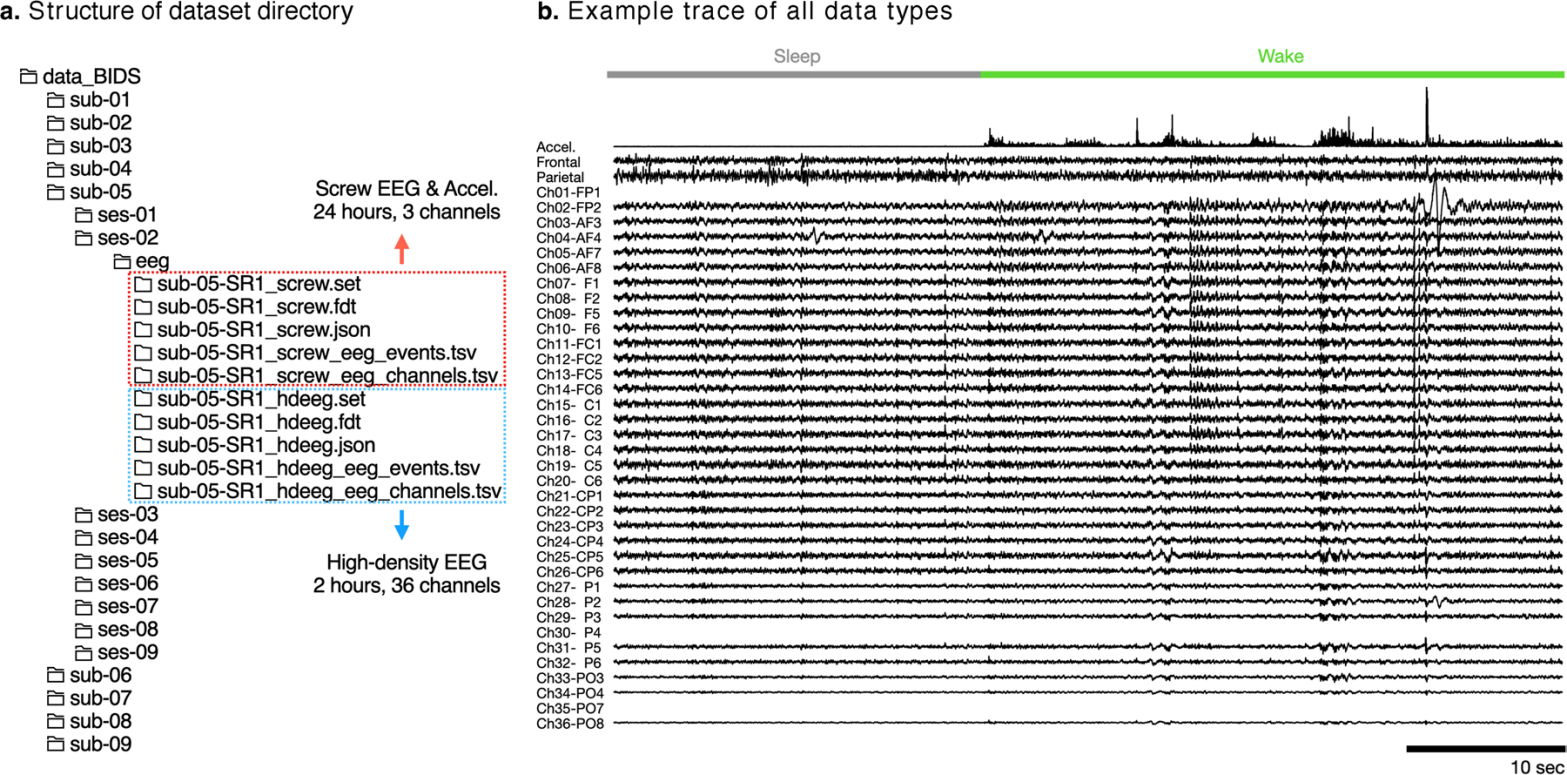
Structure of dataset. (**a**) Structure of the whole dataset organized in BIDS format. A directory of one subject (sub-**) contains 9 days of recording from one mouse, and a directory of a single session (ses-**) contains 24-hour EEG data. Single EEG data file (*.set/*.fdt) is formatted in EEGLAB-compatible format. (**b**) Example trace of all available data types, including sleep stage (top), accelerometer (1st row among black data traces), screw EEG of frontal and parietal (2nd-3rd rows among black data traces), and HD-EEG (4th to bottom rows among black data traces).

As depicted in Fig. 3a, the data files were divided into individual days. Session indicates the individual day of the experiment: the first day is baseline (BL), the second day to the sixth day is the sleep restriction period (SR1 to SR5), and the seventh to ninth days are recovery period (R1 to R3). According to the subjects and experimental days, the dataset was kept in each folder and named appropriately (ex. sub-number-experimental day data type). All datasets included a time array in the format of HH:MM:SS. 00:00:00 to signify the starting time of the daily recording, which was ZT6 in zeitgeber time.

We uploaded a tutorial written in Python to the data repository, (analysis_tutorial.ipynb) for basic handling and analysis of the files, such as file opening, basic plotting, detection of sleep-related EEG waves, and drawing power topography. The following subsections provide a detailed description of each data type, including screw EEG, accelerometer, HD-EEG, and sleep state label, as shown in Fig. 3b.

### EEG and accelerometer (continuously for 9 days)

The data are divided into days (i.e., 24 hours per file). It includes the frontal EEG (ch1, μV), parietal EEG (ch2, μV), and accelerometer (ch3, G). Data were converted to EEGLAB format (*.fdt/*.set) before they were uploaded. The time unit is in seconds. Bad channels were zero-padded as a placeholder instead of omitting them, and the information are summarized in a channel table (*_screw_eeg_channels.tsv).

### Sleep stage (selected 6 days)

Sleep were scored as wake, NREM sleep, and REM sleep, and these were assessed every 10 s using the Sleep Sign software (Kissei Comtec, Nagano, Japan). We scored the data on a daily basis, and the scored days included the baseline day, three sleep restriction days, and two recovery days, which corresponded to 1^st^, 2^nd^, 4^th^, 6^th^, 7^th^, and 9^th^ experimental day (see the data availability in Fig. 2b). The time unit is in seconds. The resulting file (*_screw_eeg_events.tsv) is included in the directory of screw EEG data.

### HD-EEG (2 hours, selected 8 days)

The HD-EEG files included 2 h of 36-channel EEG signals recorded from ZT1 to ZT3. The 2^nd^ day of recovery (i.e., 8^th^ experimental day) is missing. The units for the EEG data and time array are the same as those of the screw EEG data. Bad channels were zero-padded. The electrode labels and the bad channels in each mouse are listed in the table, *_hdeeg_eeg_channels.tsv. The event table (*_hdeeg_eeg_events.tsv) is a placeholder that contains no information.

## Technical Validation

### Signal quality of HD-EEG

**Fig. 4:**
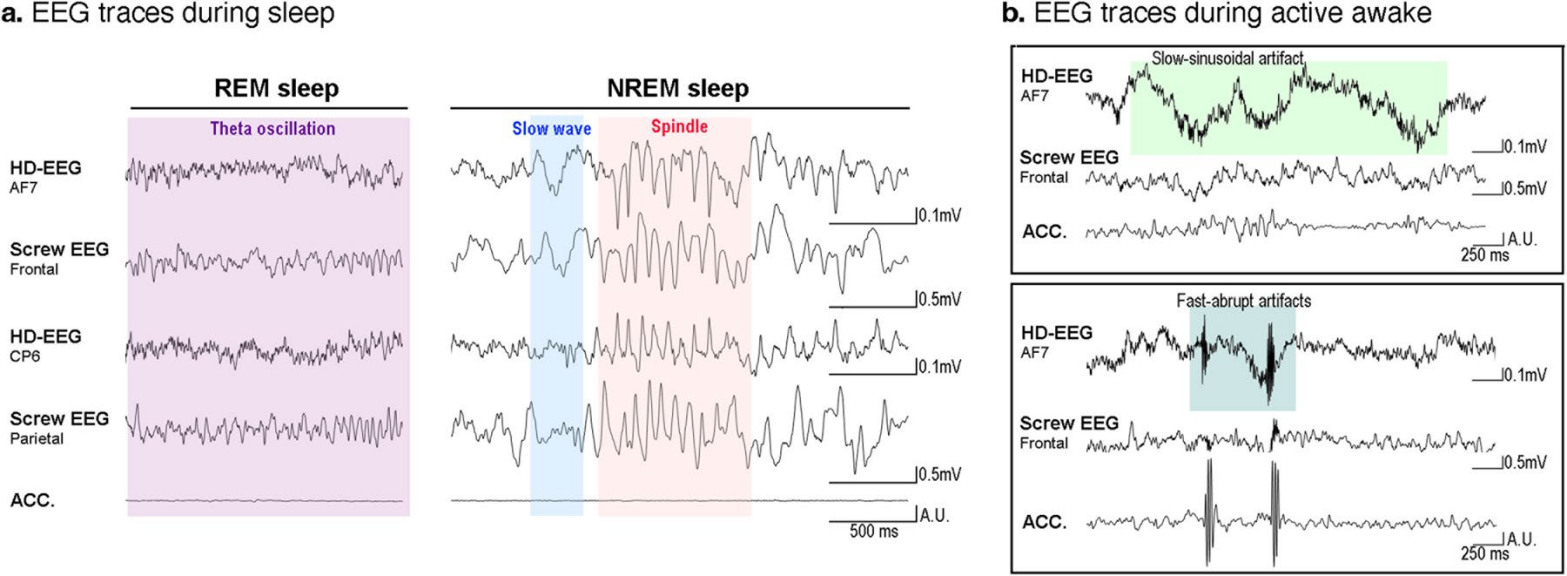
Example traces comparing HD-EEG with screw EEG. (**a**) HD-EEG (AF7), frontal screw EEG, HD-EEG (CP6), parietal screw EEG, and accelerometer traces during NREM sleep (right) and REM sleep (left), sequentially from the top. Note that distinct EEG features of sleep, which are shaded with different colors (slow wave, red; spindle, blue; theta rhythms, purple), are equally shown for screws and HD-EEG. For an accurate comparison, the polarity and size were adjusted, and HD-EEG trace of CP6 is re-referenced by AF7 for matching parietal screw EEG which is a bipolar signal between the frontal and parietal screw. (**b**) Exemplary traces of HD-EEG (AF7), frontal screw EEG, and accelerometer during active awake, which shows HD-EEGs are more vulnerable to motion artifacts than the screw EEG. The two most frequently appeared motion artifacts are shaded with light and dark green.

First, we compared the signal quality between the two-channel EEG and HD-EEG signals by comparing adjacent electrodes. Figure 4 shows an example signal of the frontal EEG and channel 5 in HD-EEG, the parietal EEG and differential between channels 21 (CP6) and 5 (AF7) in HD-EEG, and the accelerometer signal during sleep versus awake periods (see Fig. 1d for the location of HD-EEG channels). As seen in the time traces during active awake state (Fig. 4b), HD-EEGs are more vulnerable to motion compared to the screw EEG. Please refer to our previous work (Fig. 3A in^17^) to find the particular mouse motion that evokes the motion artifacts of HD-EEG. Nonetheless, we can observe similar EEG traces in the two signals during sleep. The EEG features observed during sleep (e.g., slow waves, spindles, and REM theta oscillation) were observed equally in the screw EEG and HD-EEG (Fig. 4a). Second, we checked the long-term signal quality. Previously, we showed that the signal quality was maintained over one month after surgery (see Fig. 2 in^17^). However, in our previous study, mice were tethered only during the brief recording epochs, while the mice were continuously tethered in the current work. We calculated the daily change in high-frequency power (190–250 Hz) of HD-EEG in 100 randomly selected 10-s sleep epochs. We regarded this frequency band as noise since the power of this band was high from the bad skull-electrode contact. Statistical tests over the first day showed that high-frequency power was not altered over 7 days, and it significantly increased only on the last day of the experiment (1^st^ : 0.008 ± 0.004, 9^th^ : 0.020 ± 0.013, paired t-test, p = 0.023). The signal-to-noise ratio (SNR) was monitored in two types of EEG across the day, selecting delta oscillations prominent during sleep as the signal frequency. Since delta power is altered by SD, the SNR ratio of HD-EEG to screw EEG is evaluated for comparing screw and HD-EEG with the ratio of delta power to noise. The SNR ratio displayed no statistical difference across all experimental days, which shows comparable SNR values of HD-EEG to those of skull-implanted screw EEG. The comparable signal quality of HD-EEG and its long-term stability suggest that our HD-EEG data can be a useful resource to track topographical changes of sleep EEG over a long period.

### Sleep wave and spindle analysis

**Fig. 5:**
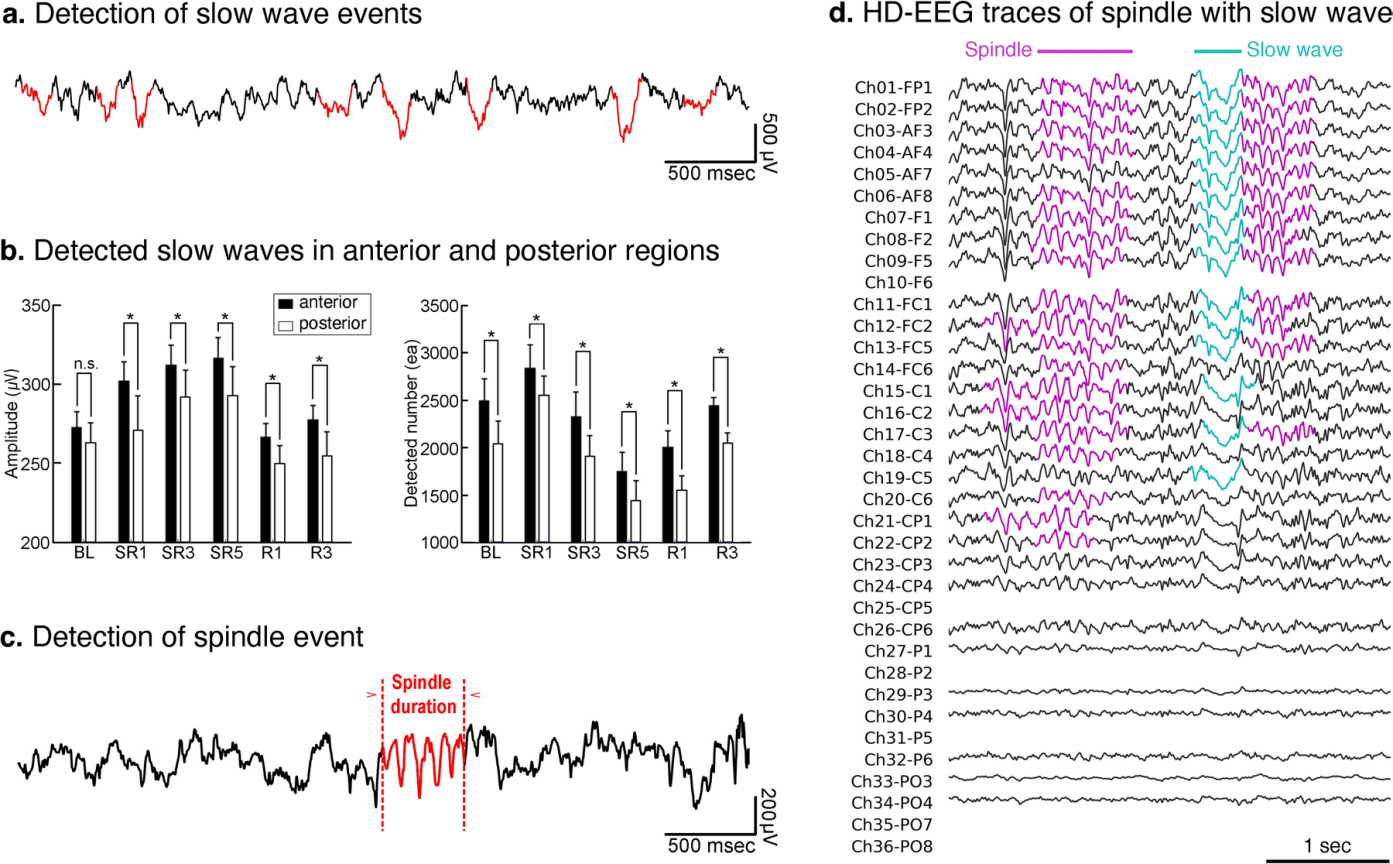
Example analyses of slow wave and spindle detection from sleep EEG dataset. (**a**) Slow wave activities (red) detected from the example EEG trace (black). (**b**) Comparisons of the amplitude and number between slow waves detected from anterior (black bar, average of Ch01–Ch10) and posterior (white bar, average of Ch13–Ch26) region for experimental days. The asterisks mark the significant differences between the two regions (paired t-test, *p < *0.05). (**c**) Example trace (black) including the detected spindle waveform (red). The spindle duration is within the range we set for spindle detection. (**d**) Example HD-EEG trace of sleep spindle event detected from Ch04-AF4. Note that the spindle follows a slow wave and that the peak positions and shapes of a spindle and slow wave are gradually differentiated in each channel of HD-EEG.

**Fig. 6:**
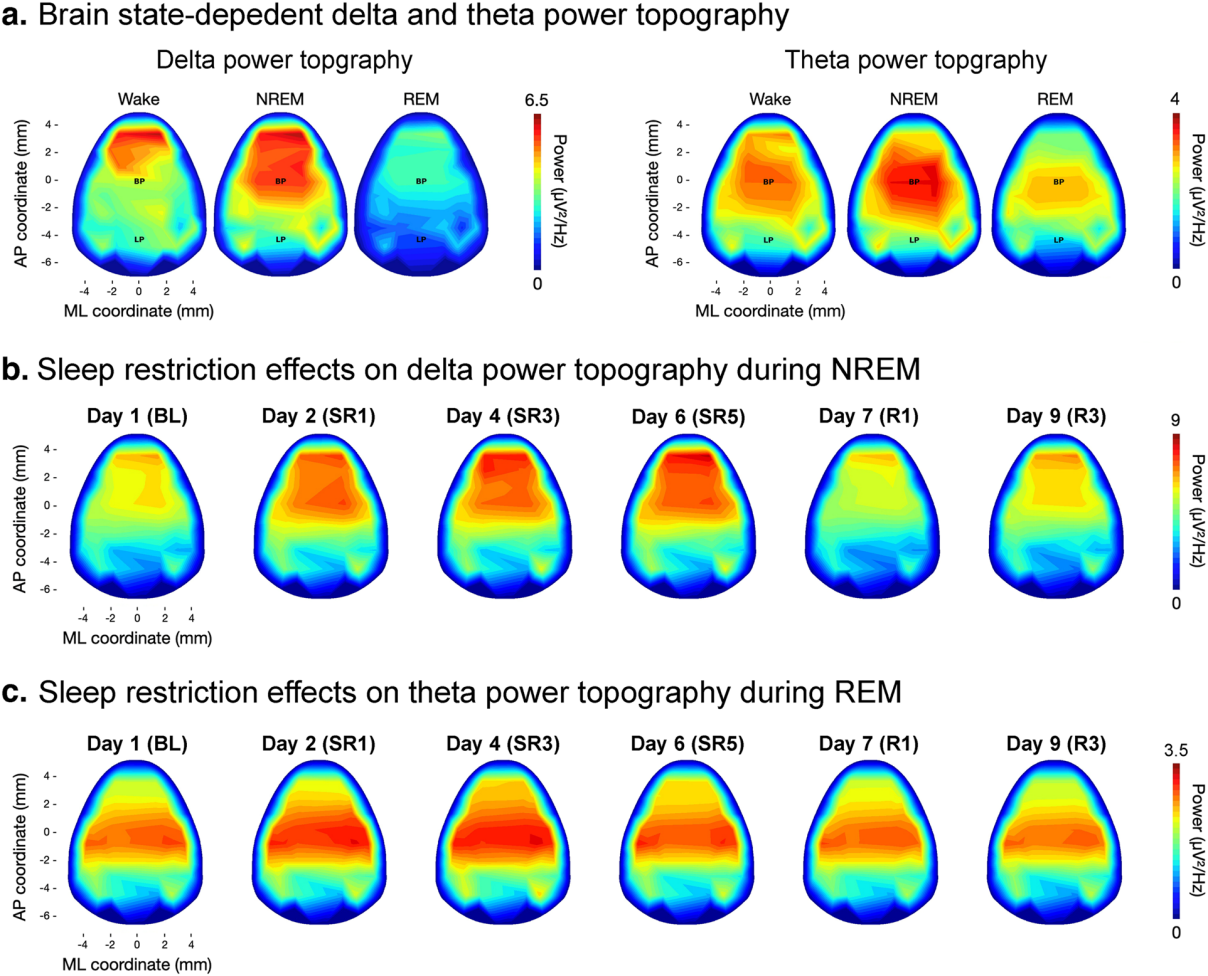
Example analyses of EEG power topography during sleep. (**a**) Example comparison of the delta (1–4 Hz) and theta (5–10 Hz) power topography over different sleep states (left: wake, middle: NREM sleep, right: REM sleep) calculated from a single session. (**b**,**c**) Demonstration of SR effect on the power topography, showing an elevated level of NREM delta and REM theta during the SR period.

Several analyses can be performed on these data to observe signal properties, such as spectral power, slow wave, sleep spindle, traveling wave, and interaction among different oscillatory activities. Here, we provide example analyses that can be performed using the dataset (Figs. 5 and 6). We used slow wave and sleep spindle as examples since these two events are the most widely studied waves in sleep EEG research. We provide Python code for detecting slow waves and sleep spindles, as well as their visualization tools to ease the reproducibility of these demonstrations. We strongly recommend the users to double-check the detected events because they are highly nonlinear.

First, the slow waves were determined by the falling and rising slopes of negative deflection in EEG and described in Figure 7B in our previous work^15^. An exemplary trace with detected slow waves is in Fig. 5a. In our previous work, we have shown that the slow waves tend to originate from the frontal cortex and propagate toward the posterior cortex (see Fig. 4B–D in^20^), similarly to the human slow wave^28^. Figure 5b compares the amplitude of slow waves in the anterior and posterior areas under different conditions, which is consistent with previous findings.

Second, the spindles were determined by the large amplitude of oscillations between 10–16 Hz and described in Fig. 2B in our previous work^20^. Our determination of spindles in HD-EEG has shown that, like in human spindles, mice have topographically distinctive sleep spindles (see Fig. 2D in^20^) and each type of spindle has different strength and latency within the cortex, thalamic relay neurons, and reticular neurons (see Fig. 3 in^20^). Figure 5c shows an example of the detected period of sleep spindle, and Fig. 5d shows the example HD-EEG traces. The first spindle was not preceded by a slow wave, but the second spindle was advanced by slow wave (cyan). In summary, HD-EEG in this work can be of a potential use for various further sleep data analyses, and the results will serve as a comparative study between human and mouse sleep.

### Topographical reconstruction of HD-EEG

The HD-EEG dataset was validated by a topographical analysis of sleep data. The Python code for the topographical reconstruction of HD-EEG was provided in our previous work. Figure 6 shows the topography of delta power during NREM and theta power during REM sleep periods under different conditions of chronic sleep restriction. It is noteworthy that both the NREM delta and REM theta powers were enhanced after the SD. NREM delta appeared dominantly in the frontal area, as in humans, whereas REM theta showed maximum amplitude in the central area of mouse topography, which suggests the different generating mechanisms of EEG oscillation in the anatomical aspect. Further detailed topographical results are presented in our previous studies on NREM^15^ and REM^16^ sleep.

## Usage Notes

We provide a nine-day continuous recording of EEG and a 2-hour HD-EEG data set as documented during the sleep restriction experiment. We performed HD-EEG recording to obtain rich spatial information of EEG in a mouse brain, and this spatial information allows many advantages for sleep EEG research. Here, we provide a few examples of research topics using this dataset.

### Investigating alterations of sleep EEG as sleep pressure accumulates

EEG rhythms during sleep may represent brain function. In many studies, it has been reported that power and regional synchrony of EEG rhythms are altered in neuropsychiatric diseases and under anesthesia^29,30^. Previously, we showed spatio-temporal specificities of several EEG rhythms, such as a frontal-dominant increase in NREM delta, and a posterior-specific increase of high theta during REM sleep, as sleep restriction repeats itself. Interesting EEG features following sleep pressure increase may be found by detailed analysis of both screw and HD-EEG.

### Spatial mapping of mouse HD-EEG could be comparable to human EEG topography

Topographic analysis is commonly used in human EEG studies, revealing circuit mechanism that generate EEG rhythms. Given that EEG oscillations and their physiological properties are conserved across species^31^, comparison of the topography of mice and that of humans may play a crucial role in translating anatomical aspects of the sleep EEG activity.

Depending on the cortical circuit involved, EEG activity shows region specificity. For example, sleep spindles have been regionally classified by slow-anterior and fast-posterior types^32,33^, and slow waves predominantly occur in the frontal cortex^28^. In addition, the relative changes in an EEG activity can be locally predominant. The region-specific dominance is believed to reflect the relatively more affected regions. Sleep homeostasis indeed has a local component, considering that the local increase in slow wave can be triggered by wake experience involving specific brain regions^34–36^. Sleep deprivation also induces regional modulation, showing frontal-dominance of slow wave increase in NREM sleep and postero-anterior gradient of alpha oscillation increase in REM sleep in humans and mice^37,38^. Cortical region-specific alterations in sleep EEG are also found in the patients with neuropsychiatric diseases^39,40^.

### Cortical information flows of sleep EEG activity in differential homeostatic sleep need

Information flow manifested in the topographical pattern of EEGs can be investigated by performing cross-regional analysis, such as cortical traveling waves and effective connectivity. Sleep is suggested to reorganize the brain for memory consolidation and restore synaptic homeostasis. The neural mechanism for these processes has been accounted for by global sleep oscillations occurring in the large-scale brain networks. Previous studies have suggested that travelling waves during human sleep spindles and slow waves could synchronize the cortical activity in a spatio-temporal manner, evoking a correlated activation between cortex and hippocampus^41–43^.

## Data Availability

All the Python scripts used for analysis in the Technical Validation section for analysis and figure generation are available in the G-Node repository.
